# Where the rubber meets the road: using FRAM to align work-as-imagined with work-as-done when implementing clinical guidelines

**DOI:** 10.1186/s13012-015-0317-y

**Published:** 2015-08-29

**Authors:** Robyn Clay-Williams, Jeanette Hounsgaard, Erik Hollnagel

**Affiliations:** Australian Institute of Health Innovation, Faculty of Medicine and Health Sciences, Macquarie University, Sydney, NSW 2109 Australia; Centre for Quality, Region of Southern Denmark, 5500 Middelfart, Denmark; Institute of Regional Health Research, University of Southern Denmark, Odense, Denmark

## Abstract

**Background:**

Uptake of guidelines in healthcare can be variable. A focus on behaviour change and other strategies to improve compliance, however, has not increased implementation success. The contribution of other factors such as clinical setting and practitioner workflow to guideline utilisation has recently been recognised. In particular, differences between work-as-imagined by those who write procedures, and work-as-done—or actually enacted—in the clinical environment, can render a guideline difficult or impossible for clinicians to follow. The Functional Resonance Analysis Method (FRAM) can be used to model workflow in the clinical setting. The aim of this study was to investigate whether FRAM can be used to identify process elements in a draft guideline that are likely to impede implementation by conflicting with current ways of working.

**Methods:**

Draft guidelines in two intensive care units (ICU), one in Australia and one in Denmark, were modelled and analysed using FRAM. The FRAM was used to guide collaborative discussion with healthcare professionals involved in writing and implementing the guidelines and to ensure that the final instructions were compatible with other processes used in the workplace.

**Results:**

Processes that would have impeded implementation were discovered early, and the guidelines were modified to maintain compatibility with current work processes. Missing process elements were also identified, thereby, avoiding the confusion that would have ensued had the guideline been introduced as originally written.

**Conclusions:**

Using FRAM to reconcile differences between work-as-imagined and work-as-done when implementing a guideline can reduce the need for clinicians to adjust performance and create workarounds, which may be detrimental to both safety and quality, once the guideline is introduced.

## Background

Uptake and implementation of clinical guidelines is variable [1, 2], with one study finding that as few as 24 % of ICU patients received complete recommended care [3]. The growing body of literature on problems related to the implementation of clinical guidelines frequently use lack of compliance as an explanation and therefore the need for behaviour change to improve this condition [4, 5].

More recent work, however, has identified organisational and other system barriers to implementation [6] finding that explanations for lack of guideline uptake more often are given at an organisational than an individual level [7]. Concentrating efforts on changing individual behaviour might therefore neither be the whole nor even the best answer to the question of how to improve uptake. Differences in organisational contexts have been shown to have a large influence on implementation success for a broad range of interventions [8]. Given that organisational context is typically fixed by resource and other constraints, it makes sense to consider adapting the intervention to the context rather than vice versa [7]. In order to do this, we need to have a clear understanding of how work is done in the clinical setting since this will never be the same as how we imagine it is accomplished [9].

In complex adaptive systems such as healthcare, [10] ‘work-as-done’ (WAD) on the front line of patient care is always different from ‘work-as-imagined’ (WAI) by those who write guidelines and procedures [11]. This can result in different or even incompatible assumptions within the organisation of how processes and tasks are accomplished and, ultimately, in introduction of written procedures that are incompatible with effective patient care.

A clinical guideline is basically a description of a series of actions or activities that are considered as necessary and sufficient to achieve a given result. It is common to think of the actions as a series of steps and/or decisions, but this takes for granted that the ordering of the actions is natural or inevitable: it describes work as it is supposed to be done (WAI). It is obvious, however, that a guideline should be realistic, hence, that it should try to correspond to work-as-done. In order to determine whether that is the case, it is necessary to be able to describe work-as-done in a concise manner.

Assumptions about how others go about their work [12] vary between providers within ICUs [13] and between hospitals [14]. For example, while some clinicians regard guidelines as a way to ensure that patients all receive the same level of care and therefore believe that the instructions should be followed explicitly, others consider guidelines more as an aspirational goal to be met or as a decision-making support for delivering high quality of care overall [14]. Developing a usable multidisciplinary procedure, therefore, can be problematic—even if it is developed in collaboration with providers and tested in the field prior to introduction. Lack of a clear, common understanding can well be the reason for poorer performance: high-performing hospitals have been shown to be more likely to discuss guidelines and their implementation with a view to sense-making and refining them to improve patient care [14].

In a study investigating compliance with guidelines in ICUs, researchers found a number of areas where lack of shared understanding led to variation in compliance [1]. This was cast in terms of ambiguities of various kinds, relating to, for instance, tasks, expectations, responsibilities, methods, and exceptions. Problems in task performance arose, for example, in processes such as ventilator weaning trials where reduction in sedation, a precursor to the task, was not accomplished by the previous shift. Guideline compliance was reduced where there was confusion about who was responsible for a given task or who was able to make a decision [1]. Method ambiguity was likely to be the result of guideline complexity in combination with the high workload demands of the ICU environment [1]. Absence of shared consensus was apparent in how conflicting priorities were addressed, especially in cases of exceptions where experience with a particular patient could contraindicate the treatment recommended in the guidelines.

While these findings suggest that improved performance and adherence to clinical guidelines is possible if only ambiguities can be reduced [15, 16], the methods for reducing ambiguity are not always clear. Many of the ‘fixes’ suggested in the literature are ‘bandaid’ fixes that patch over or work around the problem without addressing the key issue of the design of the guideline itself. One problem of a guideline that can affect uptake, for example, is how easy it is to use or apply in clinical practice [16, 17]. And for this, there is no simple ‘fix’.

While guideline characteristics, such as outdated evidence or complex statements, have been identified as barriers to implementation and compliance [18], the internal logic or consistency of the guideline and the applicability of that logic to actual workplace activities in the target health setting is rarely considered. Wherever steps in a guideline are stated implicitly rather than explicitly, there is room for differing interpretation. Whenever a guideline is written without considering the practical aspects of how work is actually done, there are likely to be inconsistencies that cannot easily be resolved in the workplace. Essential requirements to implement guidelines, such as adequate resources, are often identified only when a ‘poor uptake’ is investigated [18]. The doctors, nurses, and allied health professionals that are required to implement the new guideline are rarely considered prior to its introduction or included in the implementation process [15, 18]. Where implementation of guidelines has been most successful, the intervention has facilitated discussion among health care professionals and modification of the guidelines to meet the requirements of the health care setting [17, 19].

## Functional Resonance Analysis Method (FRAM)

The Functional Resonance Analysis Method (FRAM) [11] is a method for modelling complex socio-technical systems, which is well-suited for capturing the essential characteristics of WAD. Most methods for work analysis, including risk and safety analyses, are based on a model; the purpose of the methods is to describe the target performance in the ‘model language’, which essentially means that the target performance is mapped onto the model. FRAM is not based on a specific model of how work takes place but is rather used to produce a model that describes the functions that together make up the performance as well as their mutual dependencies. Human and organisational performance is always variable; indeed, few if any systems would function without regular patterns of approximate performance adjustments. Due to factors such as underspecified work conditions, changing environment, resource constraints, and so on, individuals must continually adjust how they work to match the changing requirements. The majority of these will lead to acceptable outcomes and therefore in hindsight be seen as ‘correct’; a few will lead to unacceptable outcomes and therefore in hindsight be seen as ‘errors’—as something that has gone wrong. Because interdependencies can be complicated and intractable, a simple tree diagram or similar is inadequate to describe system behaviour. But the interdependencies, as well as the variability, can be described by focusing on the functions that make up an activity.

**Fig. 1:**
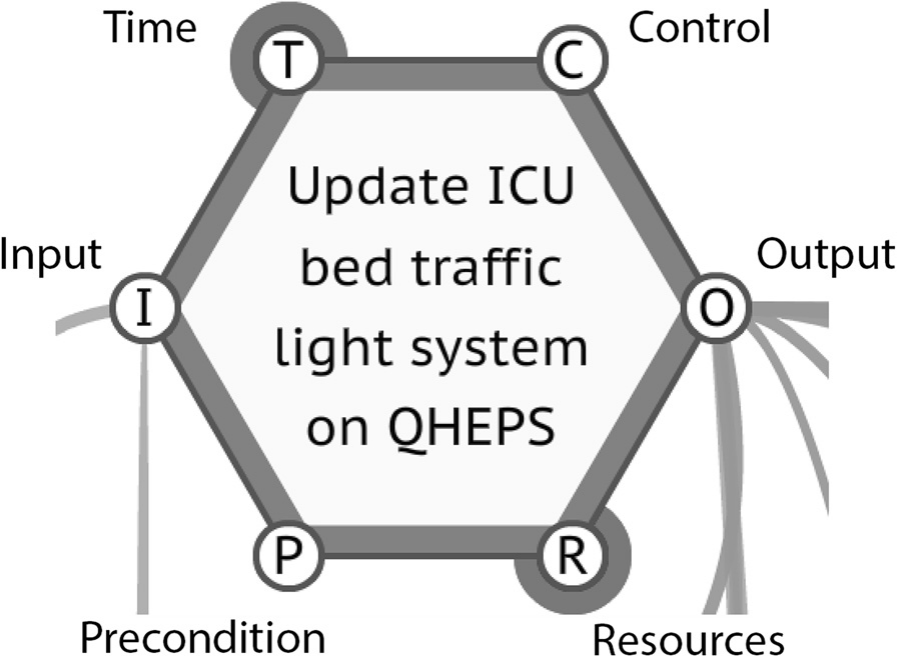
FRAM activity hexagon

A FRAM model is developed by determining the activities or functions that make up a process and how they are coupled. The data for developing a FRAM model can be obtained through a number of methods, including ethnography, interviews, documented processes, and so on. Each function is then described in terms of its aspects (see Fig. 1):

1. Input (I) is what the function acts on or changes (an input is also used to start the function).
2. Output (O) is what emerges from the function (this can be an outcome or a state change).
3. Precondition (P) is a condition that must be satisfied before the function can be commenced.
4. Resources (R) are materials or people needed to carry out the function or material consumed during the function.
5. Control (C) is how the function is monitored or controlled.
6. Time (T) refers to any time constraints that might affect completing the function.

In relation to guidelines, the FRAM can be used to describe the written guideline as a set of functions together with the conditions that are necessary for the realisation of the functions. The resulting model can be used to analyse the potential variability of conditions and functions and how this may combine to affect the way in which the guideline is implemented.

A FRAM model is built using a software tool called the FRAM Model Visualiser (FMV) [20]. One feature of the FMV is that it identifies conditions that are incompletely described and marks them graphically (see Fig. 1, bold circles). In the case of modelling a guideline, this makes it easy to find the parts of the guideline that are incompletely described or not described at all.

The logic of the FRAM is used to develop questions that are then explored with those who will be using the guideline in the workplace. Typical questions that might be explored are illustrated in Table 1.

**Table 1:** Guided questions for exploring the FRAM conditions

Condition	Guided question
Input	What starts the function?
	What does the function act on or change?
Output	What is the output or results of the function?
	Do you have to inform anyone?
	Do you have to collect or record/report anything? If so, where?
	Who needs the output? Who will use what is produced? Have you agreed with whoever uses this that it is what they need?
Precondition	What should be in place so that you can complete the function normally?
	What do you do if the preconditions are not available?
Resource	What resources do you need to perform the function, such as people, equipment, IT, power, buildings, etc?
	What do you do if the resources are not available?
Control	Do you have any goals for the function, such as do something within a time frame (this is a control)?
	What is the purpose of this function? Why do we do this?
	Do you have formal procedures or instructions controlling the function?
	Do you have people, such as supervisors, controlling the function?
	Are there values controlling the function?
	Do unofficial work practices or culture control the function?
	Do you have priorities, such as a triage system?
	Are there constraints such as budget?
Time	Is there any time related to the function?
	Is there a certain time where you have to perform the function?
	What happens if you are delayed—will you still do the function or not and what is the consequence for the following functions?
	Time only has four options: too early, too late, on time, or not at all.

Procedures are usually created in isolation, away from the ‘sharp end’, with little if any consideration of how they fit processes that already are in place. The FRAM can be used to identify the functions that a procedure describes as well as how they depend on each other and on external conditions. By identifying the dependencies between functions, the model can be used to show how the new procedure can be accomplished in this ‘real’ world. In effect, the FRAM can be used to determine whether it is *possible* to follow a procedure as written or published and can thus be the basis for finding practical solutions to problems thus found. The FRAM makes it possible to identify differences between WAD and WAI and to resolve ambiguities in guidelines. In this way, it offers a practical approach to tackle the fundamental problems encountered in procedure design and implementation.

## Methods

To demonstrate how FRAM might be used to improve guideline development and implementation, we looked at how FRAM could be used to gain insight into the practicality of a new procedure. We chose two ICU guidelines for this purpose.

### Case1: intra-hospital transfer of critically ill patients

The first guideline was developed in an ICU in Denmark to resolve a conflict between the ICU and the medical ward about the transfer of ICU patients to the ward. Intra-hospital transfer can pose risk to critically ill patients, and adoption of guidelines is recommended to improve safety [21]. Medical ward clinicians were concerned that patients were being transferred from the ICU before they were sufficiently recovered to allow safe transport thereby increasing workload for the ward staff members. The ICU, however, felt that the ward did not allocate sufficient resources for safe transfer and that it did not prepare well enough to transition the patient from high to low monitoring. Relatives of the patients were concerned that their family member would receive a lesser level of care with the reduced monitoring; ICU staff felt that the ward staff did not give sufficient weight to this distress.

The clinicians at the ICU and the ward met, hoping to solve the conflict and to find possibilities for improving the transfer through dialogue. Both practitioners and nurses from the two units, together with a ward quality manager, participated in the meeting, which was facilitated by a researcher experienced in FRAM model development (JH). Although none of the clinicians had prior knowledge of the FRAM, they were convinced, after a short introduction, to use the method to describe work-as-done. The researcher guided the group in describing the written guideline as a set of functions with associated conditions and through the process of developing the FRAM.

### Case 2: managing demand for clinical services in the ICU

The second guideline, consisting of an ICU bed escalation plan and instruction, was developed for implementation in an ICU in regional Australia. The objective of the guideline was to ensure that the increasing demands for clinical services of the ICU were managed in a way that allowed improved planning for elective cases while managing the unpredictable nature of emergency admissions. Frequent cancelling of elective surgery at short notice, due to lack of ICU beds, had resulted in distrust between the surgery department and ICU. The guideline was written to lay down rules underpinning ICU decisions on availability of beds for elective surgery patients and to repair relationships between the two departments. The plan proposed three readiness states for the ICU. A state of GREEN indicates that the unit is comfortable with patient load, and the booking of ‘extra’ elective surgery cases may be considered. At a state of AMBER, the ICU is approaching full; given the expected unplanned and elective admissions in the next 24 h and taking into account expected discharges, the unit will be at capacity. A state of RED indicates that the unit is at capacity; all on-call nursing staff members have been used, all safe patient discharges have occurred or the hospital is physically full, and no discharge beds are available. At RED, elective surgery cases are cancelled.

### Common processes

Superficially, both procedures were well written and appeared to be ready for implementation. For each guideline, an initial FRAM model was constructed based on the original written guideline. The results were used to guide discussion with ICU clinicians and to identify areas where WAD was likely to differ from WAI. A version of the guideline that aligned better with WAD was then developed, via an iterative collaboration between researchers and clinicians, and implemented in the ICU.

## Results

### Case 1: intra-hospital transfer of critically ill patients

**Fig. 2:**
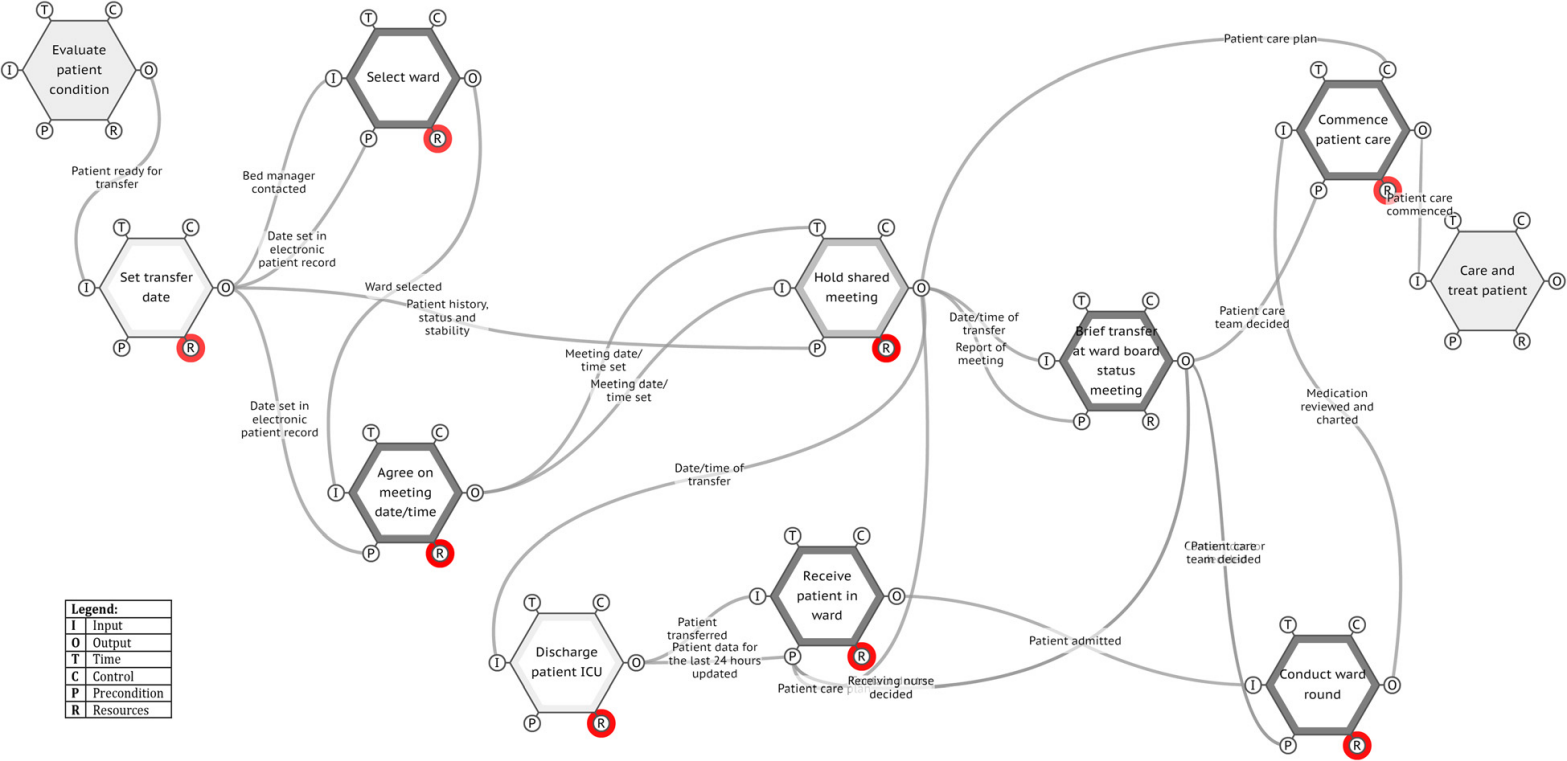
FRAM of intra-hospital transfer protocol

The clinicians spent 1.5 h in the meeting developing a FRAM model of work-as-done (WAD). The FRAM analysis identified nine functions, which were entered into the FMV (Fig. 2). The main purpose of the guideline was to make patients and relatives feel secure with the patient’s transfer from ICU to the medical ward, by ensuring that the transfer was coordinated and aligned between staff members, the patient and their relatives. The FRAM model showed that no functions were defined to support this primary purpose. From the FRAM model, it became apparent that the common meeting called at short notice and involving a doctor and a nurse from both departments was likely to be impractical. This was not only due to problems coordinating staff attendance but also because other functions, such as collating data on health status and stability of the patient and drug reconciliation were preconditions for a successful outcome. In addition, the analysis showed that many other functions would depend on the successful output of the common meeting; this can easily be seen by the graphical representation of the couplings. For example, the discharge function of the ICU would need the exact discharge date; the receiving of the patient in the medical ward would need the patient care plan to succeed; the briefing of the staff at the medical ward would need both the actual date of transfer and also the information from the common meeting as a precondition for success; and finally, to commence patient care in the medical ward, the patient care plan was needed to control the function. On reflection, the clinicians felt that the function of a common meeting was unrealistic, and they decided to consider other solutions. FRAM also raised other important issues that had not been considered, such as the following: Who has the authority to set the actual date for transfer? How is the information from the common meeting brought to the knowledge of the medical ward? And how can the ward in cooperation with the ICU handle a patient getting worse safely, avoiding a readmission to the ICU?

The clinicians easily understood the FRAM model, showing how they imagined the future transfers to be, even though it was only the second time they had encountered FRAM. Experiences from previous work with FRAM in a clinical setting in Denmark showed that the FRAM thinking, structure and model are easily understood by clinicians. Reactions from clinicians included the following: ‘This is the first time, I have used an analysis method that shows how things really work’, ‘I will join again, if this is the way we are going to analyse in the future’ and ‘FRAM gives us common understanding of what is really going on’. Experiences of one researcher (JH) from previous work with clinicians also showed that clinicians quickly can learn to use the FRAM without the assistance from researchers or developers.

### Case 2: managing demand for clinical services in the ICU

Rather than assembling a team in clinicians, in this case the initial FRAM was developed by one researcher (RCW) from the written guideline. Each activity in the guideline, and any associated conditions, was identified and entered into the FMV. The guideline was comprised of 22 functions. An example of one of the activities, and its associated conditions, is in Table 2.

**Table 2:** Example of ICU guideline function

Function: plan actions for next day	
Condition	Description
Input	Staff meet
Output	Actions for next day
	Agreement (yes/no) to start first heart surgery at 0700
	Any hold actions beyond first heart surgery until meeting 0800
Precondition	Bed plan
	Discharge plan for next day
	Authorisation for transfer of overflow
	Number of nurses available
Resource	Nurse Unit Manager
	ICU General Consultant
	ICU Surgical Consultant
	Nursing Director
	Surgical Services Group representative
Control	ICU capacity to accept unplanned admissions within the next 24 h
	Ability to accept elective workload
	Number of elective surgeries next day
	Prioritisation of cases
Time	1600 (meeting time)
	0700 (output required)
	0800 (output required)

On examination, many of the functions, particularly in the RED state, had outputs that were not used by other functions and there were few couplings between functions. Consequently, if the guideline were to be followed exactly as written, a number of the activities could not be completed. From the FRAM, it was evident that some functions were common to all states and also that some intended functions, such as releasing beds for additional elective patients in the GREEN state, were missing. The problems were not evident in the written guideline and only became obvious once the FRAM model was built.

**Fig. 3:**
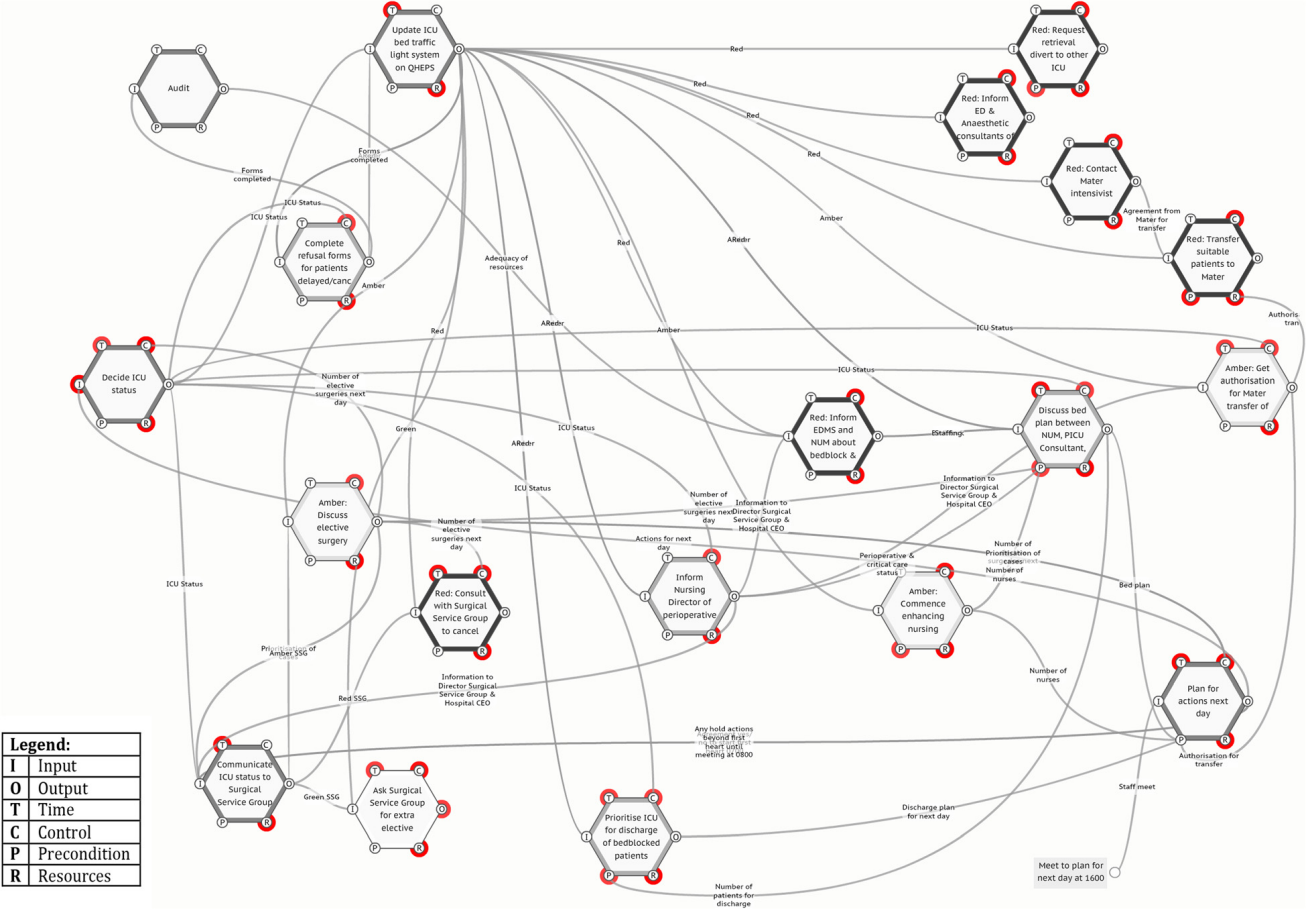
FRAM of modified guideline draft

The resulting FRAM model was discussed with the senior ICU consultant, senior nursing staff and surgical staff to resolve the ambiguities. For the GREEN state, three new functions were added: one to update the traffic light system on the central computer and two to inform surgery of spare ICU capacity. The bed planning meeting and ICU action planning meetings were common for all three conditions, so were combined. As a result, a second FRAM model was constructed (Fig. 3). This model comprised 20 functions, including addition of items that were missing from the original guideline, and began to show a closer alignment with expected WAD. As would be expected in a complex workplace, additional dependencies appeared on many functions. Functions with high numbers of dependencies were flagged as being likely to be more difficult to accomplish in the real world of everyday work than those with fewer dependencies. The bed planning meeting function, for example, had a high number of dependencies and required clinicians from the ICU, surgery and the hospital bed management team to meet at the same time each day to develop a bed plan for the following day. On reflection, the ICU clinicians felt that this was unrealistic given the workloads and schedules of the meeting participants, and they agreed to accomplish this task via other means.

In each of these cases, FRAM development meetings were conducted over a few weeks to accommodate the work schedules of the participants. The process was not cumbersome, however, and could be compressed to less than a day if priority for implementation was high.

## Discussion

The FRAM turned out to be an excellent tool for initiating discussion with providers on how to implement written guidelines. The visual representation of the functions, automatically produced by the FMV, was easy to understand, and the couplings provided a useful visual representation of the number of interdependencies between tasks. Likely ‘sticking points’ were easily identified, as were areas that did not make sense in actual clinical practice. For the Danish guidelines, the resulting model showed that there were no functions in the two units to support coordination of the transfer, cooperation between ICU and ward staff or communication with the patients and their relatives. The group concluded that the guideline would never succeed if they continued their practice without changes. In the Australian guideline, there was no function to alert surgeons that additional ICU beds were available.

The real work in modifying the guidelines, however, was accomplished during the collaborative meetings between the clinicians; the FRAM was merely a tool to facilitate these discussions. The Danish clinicians also acknowledged that no one was to blame—the existing organisation of the work did not support a successful transfer of patients from ICU to ward. The FRAM provides feedback that can be used to iterate and refine the procedure or guideline prior to introduction. After the meeting in Case 1, the Danish clinicians at the ICU and the ward together developed a usable guideline to support future transfers. The presence of an active feedback system has been found, in combination with shared mental models, to be a marker of high-performing organisations [14] and improves adherence to guidelines once they are in place [17, 18].

As a result of the FRAM analyses, both ICUs realised that their first proposal was not feasible and both concluded that they had to re-design the guidelines. In both cases, there were elements that clinicians wanted to include, but since these were missing from the original guidelines, they had to find alternate ways of achieving those goals. Also both guidelines depended on common meetings called at short notice that on second thought were deemed impractical. Part of the strength of this method is that it allowed clinicians and leaders to identify the problems with a proposed guideline themselves, without the need for expensive and time-consuming external support.

### Study limitations

When aligning WAI with WAD in implementing guidelines, it is important to identify any mandatory requirements. In both of our case studies, it was appropriate to modify the guideline to meet the needs of the clinicians. This was necessary because the guidelines would not have been actionable if they had been implemented in their original form. In some cases, however, such as implementation of evidence-based clinical guidelines, it may be necessary to modify the work practices of the clinicians to correspond better to the needs of the guideline. A FRAM may still prove useful in identifying what needs to change for implementation to be effective, but the thing that needs to change might be an aspect of clinician behaviour.

## Conclusions

Guidelines provide direction for WAI, but if they do not take into account the realities of WAD, they are unlikely to be effective. At present, if this problem is addressed at all, it is usually solved by workarounds. These may be either explicit, such as posted notes or visual reminders, or implicit such as clinicians ignoring the guidelines in favour of alternate procedures [1]. FRAM provides a method for identifying the differences between WAI and expected WAD and for modifying the guideline accordingly prior to introduction into the workplace. Therefore, rather than having the facility wasting resources on changing behaviour of providers, the guideline can be introduced in a form that is already compatible with how providers are working.

The significant benefit of a FRAM analysis is that the guideline is not just modified cosmetically but that it is reformulated to reconcile WAI and WAD. One consequence of that is a reduced need to adjust performance and create workarounds that may be detrimental to both safety and quality. Another consequence is that the need of clinicians to second-guess what the facility had in mind is reduced to a manageable level.

## Abbreviations

FMV: FRAM Model Visualiser
FRAM: Functional Resonance Analysis Method
ICU: intensive care unit
WAD: work-as-done
WAI: work-as-imagined
